# Pretest expectations strongly influence interpretation of abnormal laboratory results and further management

**DOI:** 10.1186/1471-2296-11-13

**Published:** 2010-02-16

**Authors:** Paul HH Houben, Trudy van der Weijden, Bjorn Winkens, Ron AG Winkens, Richard PTM Grol

**Affiliations:** 1 Maastricht University, School of Public Health and Primary Care (CAPHRI), Department of General Practice, PO Box 616, 6200 MD Maastricht, the Netherlands; 2 Maastricht University, School of Public Health and Primary Care (CAPHRI), Department of Methodology and Statistics, PO Box 616, 6200 MD Maastricht, the Netherlands; 3 Maastricht University Medical Centre, Integrated Care Unit, PO Box 5800, 6202 AZ Maastricht, the Netherlands; 4 Radboud University Nijmegen, Scientific Institute for Quality of Healthcare, PO Box 9101, 6500 HB Nijmegen, the Netherlands

## Abstract

**Background:**

Abnormal results of diagnostic laboratory tests can be difficult to interpret when disease probability is very low. Although most physicians generally do not use Bayesian calculations to interpret abnormal results, their estimates of pretest disease probability and reasons for ordering diagnostic tests may - in a more implicit manner - influence test interpretation and further management. A better understanding of this influence may help to improve test interpretation and management. Therefore, the objective of this study was to examine the influence of physicians' pretest disease probability estimates, and their reasons for ordering diagnostic tests, on test result interpretation, posttest probability estimates and further management.

**Methods:**

Prospective study among 87 primary care physicians in the Netherlands who each ordered laboratory tests for 25 patients. They recorded their reasons for ordering the tests (to exclude or confirm disease or to reassure patients) and their pretest disease probability estimates. Upon receiving the results they recorded how they interpreted the tests, their posttest probability estimates and further management. Logistic regression was used to analyse whether the pretest probability and the reasons for ordering tests influenced the interpretation, the posttest probability estimates and the decisions on further management.

**Results:**

The physicians ordered tests for diagnostic purposes for 1253 patients; 742 patients had an abnormal result (64%). Physicians' pretest probability estimates and their reasons for ordering diagnostic tests influenced test interpretation, posttest probability estimates and further management. Abnormal results of tests ordered for reasons of reassurance were significantly more likely to be interpreted as normal (65.8%) compared to tests ordered to confirm a diagnosis or exclude a disease (27.7% and 50.9%, respectively). The odds for abnormal results to be interpreted as normal were much lower when the physician estimated a high pretest disease probability, compared to a low pretest probability estimate (OR = 0.18, 95% CI = 0.07-0.52, p < 0.001).

**Conclusions:**

Interpretation and management of abnormal test results were strongly influenced by physicians' estimation of pretest disease probability and by the reason for ordering the test. By relating abnormal laboratory results to their pretest expectations, physicians may seek a balance between over- and under-reacting to laboratory test results.

## Background

Laboratory tests are frequently ordered in routine primary care as part of the diagnostic process, even though the physician's pretest expectation may often be that the probability of disease is low, and they often order tests for other than purely medical reasons, such as patient reassurance [1,2]. As a consequence of the statistical definitions used for the reference values for laboratory tests, abnormal results are frequent, even in healthy individuals [3]. For example, in a screening programme with healthy individuals, a battery of 8 blood chemistry tests yielded at least one abnormal result for 20.6% of the individuals [4]. Abnormal results may therefore be difficult to interpret, certainly in the light of the low probability of serious disease in the primary care population [5].

When physicians interpret test results and make plans for further management, it would be interesting to know to what extent they take their pretest expectations into account. For example, many physicians have difficulty performing Bayesian calculations to interpret test results, as such calculations may be complex and are often not easily applicable to situations where several diagnostic hypotheses are considered and several tests are ordered [6]. However, physicians' pretest expectations, such as their estimates of pretest probability and their reasons for ordering diagnostic tests (for example to exclude or confirm disease or to reassure patients) may influence test result interpretation and management, though perhaps not in a direct Bayesian fashion but probably in a more implicit way. Although the influence of physicians' pretest expectations on the ordering of tests has been extensively studied, little is known about the influence of their pretest expectations on the interpretation of test results and further management in routine care, as research on this subject has been scarce [7,8]. A better understanding of this influence may help to improve test interpretation and management. The objective of this study was therefore to examine the influence of physicians' pretest expectations in terms of estimated pretest disease probability, and their reasons for ordering diagnostic tests, on the subsequent interpretation of the results of these diagnostic tests and further management.

## Methods

### Design and Setting

We conducted a prospective study among primary care physicians and their patients in 7 rural, suburban and urban areas in the south of the Netherlands, in 2004/05. Each participating physician was instructed to record data on 25 adult patients for whom they had decided to order laboratory tests during the consultation. To prevent selection bias, they were instructed to include the first 25 patients for whom laboratory tests were ordered, without any further selection. Physicians working part-time included a smaller number of patients, proportional to the number of hours a week they worked. Patients were asked to give informed consent. The Maastricht Medical Ethics Committee approved the study (reference number MEC 03-195-1).

### Measurements

The physicians recorded data both when they ordered the laboratory tests and when they received the test results, using forms that were specifically designed for the study and took about 2 minutes to complete [additional file 1] [additional file 2]. The forms had been pilot-tested and evaluated as regards validity, reliability and user convenience in an iterative process among a sample of ten primary care physicians and a questionnaire expert.

### Variables, pretest expectations

#### Reason for ordering tests

We distinguished nine reasons for test ordering, which were chosen on the basis of a qualitative interview study among primary care physicians about ordering and interpreting laboratory tests [9]. Physicians recorded the most important reason for ordering the investigations by ticking one of nine check-off boxes. We summarized these into five categories: (1) to exclude disease and reduce the physician's own uncertainty, (2) to confirm diagnosis and to determine treatment, (3) to reassure patients and at patients' request, (4) to screen for hypertension/cholesterol/diabetes and check-up for a known disorder and (5) other reasons.

#### Pretest estimate of disease probability

The form that the physicians had to complete asked: 'Do you suspect that the patient has a disease?'. The physicians answered on a 5-point Likert scale: 'definitely not', 'probably not', 'maybe', 'probably yes' and 'definitely yes'.

### Variables, outcomes

#### Interpretation of the laboratory results

When the physicians received the results we asked them: 'How do you interpret these results for this patient?' The physicians answered on a 3-point scale; 'normal', 'possibly abnormal' or 'clearly abnormal'.

#### Posttest estimate of disease probability

The form that the physicians had to complete asked: 'Do you suspect that the patient has a disease?'. The physicians answered on a 5-point Likert scale: 'definitely not', 'probably not', 'maybe', 'probably yes' and 'definitely yes'.

#### Management

We distinguished nine management items, and physicians were instructed to select one or two items in check-off boxes. We classified these items into passive and active management items. Passive management items were 'reassurance/explanation', 'expectative/wait-and-see', 'advice (about lifestyle, complaints, etc.)', and 'instructions'. Active management items were 'additional investigations (laboratory, imaging, etc.)', 'new/follow-up appointment', 'medication (start, stop, change)', referral (specialist, other health care provider) and 'other management'. We defined the management as active if at least one of the checked boxes was an active management item.

### Analysis

Only patients for whom the physician ordered laboratory tests for diagnostic purposes (reasons 1-3) were included in the analysis. We excluded patients for whom tests were ordered for screening, check-up or other reasons. We included all test results reported by the regional laboratories and defined a patient's results as abnormal if at least one test was outside the laboratories' reference values. We used chi-square tests to identify significant differences in interpretation, posttest probability estimates and management between the various reasons for ordering tests and between the various estimates of pretest probability.

Three logistic regression models were applied to the data. The first model analysed the influence of patients' age and sex, the reason for ordering tests and the pretest probability on the interpretation of the results. The second model incorporated the previous variables, plus the interpretation of the results, as independent variables, and analysed their influence on the posttest probability estimates. Finally, the third model investigated the influence of all previous variables on the management. To be able to apply logistic regression, we dichotomized the dependent variables, distinguishing the categories 'normal' and 'possibly or clearly abnormal' for the interpretation of results, and the categories 'low probability' (definitely not/probably not) and 'high probability' (maybe/probably yes/definitely yes) for the posttest probability. We considered P-values smaller than or equal to 0.05 to be significant. We checked for multicollinearity (condition index >30 and variance decomposition proportion (VDP) > 0.5) and tested the goodness-of-fit using the Hosmer and Lemeshow test. Also, to check whether the responses obtained for the individual patients were correlated within doctors, we performed a generalized estimating equations (GEE) analysis with an exchangeable working correlation structure. All analyses were performed with SPSS version 16.0.

## Results

Eighty-seven primary care physicians participated, and together they included 1775 patients (table 1). Laboratory tests were ordered for diagnostic reasons for 1253 (71%) patients. We received no laboratory results for 7.2% of these patients, the primary reason being that patients failed to visit the laboratory (29%). The laboratories reported 11,548 tests for the remaining 1,163 patients, a mean of 9.9 tests per patient. The most common reason for ordering tests was to exclude disease (62%). Tests for reassurance were ordered for 20% of the patients. The estimated pretest disease probability was low for 43% of the patients (table 2). There were 742 patients (64%) with a laboratory result including one or more abnormal tests.

**Table 1:** Characteristics of primary care physicians and patients

Primary care physicians	N = 87 (NIVEL*)
Sex	
man	68% (67%)
woman	32% (33%)
	
Age	
<50 years	68% (55%)
>50 years	32% (45%)
	
Experience **	
<15 years	41%
>15 years	59%
	
Working	
full-time	52% (54%)
part-time	48% (46%)
	
**Patients**	**N = 1253**
	
Sex	
man	38%
woman	62%
	
Age	
18-40 years	33%
40-60 years	37%
60+ years	30%

* the Netherlands Institute for Health Services Research http://www.nivel.nl documents data on all Dutch primary care physicians (N = 8408, data 2005).

** No Nivel data available

**Table 2:** Reasons for ordering laboratory tests and pretest probability estimates

*Reason for ordering lab tests*	N = 1147 (16 missing)
reassure patient	226 (20%)
exclude disease	708 (62%)
confirm diagnosis	213 (19%)
	
*Estimate of pretest disease probability*	N = 1138 (25 missing)
definitely no disease	114 (10%)
probably no disease	377 (33%)
maybe	329 (29%)
probably disease	252 (22%)
definitely disease	66 (6%)

The physicians interpreted the abnormal laboratory results for these 742 patients as normal in 48% of the cases, while their estimation of the posttest probability was low in 49.5% of the cases, and their management consisted of 'no action' for 49.2% of these patients. The percentage of patients whose abnormal results were interpreted as normal was significantly larger if tests were ordered to reassure (65.8%) compared with other reasons (50.9% and 27.7%, p < 0.001) and was significantly larger if the pretest probability was estimated to be low (66.1%) compared with high pretest probabilities (19.6%, p < 0.001). Similar significant relations were found for the posttest probability estimates and the management (table 3). If tests were ordered for reassurance or if the physicians' pretest probability estimate was low, the interpretation for patients (comparable in terms of age and sex) having only normal results was 'normal' in 100% of the cases. The posttest probability estimates were low in 100% and 96.6% of the cases, respectively, while the management was 'no action' in 88.9% and 91.2% of the patients, respectively.

**Table 3:** Interpretation, posttest disease probability estimates and management after receiving abnormal laboratory results.

	Test interpretation	Posttest probability	Management
	= normal	= no disease	= no action
*Reason for ordering lab tests*			
reassure patient	75 (65.8%)*	90 (76.9%)*	85 (75.2%)*
exclude disease	220 (50.9%)	224 (51.1%)	214 (49.4%)
confirm diagnosis	41 (27.7%)	34 (23.0%)	43 (28.9%)
			
*Pretest probability estimate*			
definitely no disease	37 (66.1%)*	48 (84.2%)*	42 (76.4%)*
probably no disease	139 (67.1%)	150 (71.8%)	137 (66.8%)
maybe	95 (45.2%)	101 (47.6%)	91 (43.1%)
probably disease	55 (31.4%)	43 (24.0%)	58 (32.8%)
definitely disease	9 (19.6%)	6 (13.0%)	14 (29.8%)

*Chi-square test, p < 0.001

If tests were ordered for reassurance, the percentage of patients with abnormal results being offered further diagnostic investigations was 8.8%, while none of the patients whose laboratory results were normal were offered further investigations. Of the patients with a low pretest probability and abnormal results, 11.1% were offered further investigations by their physician, while 3.7% of the patients with a low pretest probability and normal results were offered further investigations.

Table 4 shows the results of the logistic regression analysis. There was no multicollinearity. Compared to a low pretest probability estimate, a high estimate decreased the likelihood that abnormal results were interpreted as normal (OR = 0.18, 95% CI = 0.07-0.52, p < 0.001) and also decreased the likelihood of a low posttest probability estimate (OR = 0.04, 95% CI = 0.01-0.23, p < 0.001). The physicians were also less likely to interpret abnormal tests as normal if the laboratory tests were ordered to confirm a diagnosis, compared to those ordered to exclude disease (OR 0.59, CI 0.37-0.93, p = 0.067). The likelihood of passive management ('no action') increased if tests were ordered for reassurance, compared to those ordered to exclude disease (OR 2.25, CI 1.08-4.66, p = 0.06).

**Table 4:** Influence of pretest expectations on interpretation, posttest probability estimates and management after abnormal results.

	Test interpretation	Posttest probability	Management
	= normal^†^	= no disease^‡^	= no action^§^
	odds ratio (95% CI)	odds ratio (95% CI)	odds ratio (95% CI)
*Reason for ordering lab tests*	p = 0.067	p = 0.52	p = 0.06
exclude disease	1	1	1
reassure patient	1.1 (0.63-1.83)	1.46 (0.67-3.17)	2.25 (1.08-4.66)
confirm diagnosis	0.59 (0.37-0.93)	0.82 (0.42-1.59)	0.80 (0.46-1.40)
			
*Pretest probability estimate*	p < 0.001	p < 0.001	p = 0.19
definitely no disease	1	1	1
probably no disease	1.13 (0.56-2.26)	0.35 (0.11-1.08)	1.67 (0.64-4.32)
maybe	0.49 (0.24-1.03)	0.20 (0.06-0.63)	1.11 (0.41-3.00)
probably disease	0.33 (0.15-0.73)	0.07 (0.02-0.23)	1.75 (0.59-5.17)
definitely disease	0.18 (0.07-0.52)	0.04 (0.01-0.23)	2.88 (0.79-10.59)
			
*Test interpretation*	N/A*	p < 0.001	p < 0.001
normal		1	1
possibly abnormal		0.09 (0.06-0.15)	0.20 (0.10-0.40)
abnormal		0.01 (0.005-0.02)	0.43 (0.25-0.72)
			
*Posttest probability estimate*	N/A*	N/A*	p < 0.001
definitely no disease			1
probably no disease			0.48 (0.26-0.89)
maybe			0.14 (0.07-0.32)
probably disease			0.07 (0.03-0.16)
definitely disease			0.09 (0.04-0.23)

N = 742

*N/A not applicable

^†^Goodness-of-fit: Chi-square = 5.29, p = 0.73, ^‡ ^Chi-square = 7.12, p = 0.52, ^§ ^Chi-square = 5.70, p = 0.68

The intraclass correlations calculated by the generalized estimating equations analysis were small. For the three models they were 0.022, 0.050 and 0.015 respectively. There were similar results for the significance of the variables and the odds ratios as compared to the results from the logistic regression analysis.

## Discussion

The results show that the interpretation of test results, posttest disease probability estimates and management were significantly influenced by the physicians' pretest expectations. If the pretest probability was low or when tests were ordered at patients' request or to reassure them, the physicians tended to interpret abnormal results as normal and not to initiate further action. On the whole, this may be a correct decision, since many laboratory abnormalities will not be clinically relevant if the pretest probability is low. Physicians may use their pretest expectations to seek a balance between over- and under-reacting to laboratory test results.

To our knowledge, research about physicians' routine interpretation of laboratory results is still scarce. This study attempted to examine what they do with the results of laboratory tests. Strong points of this study were that it included many physicians and patients, that the data were prospectively collected and that we tried to prevent selection bias by instructing the physicians to include consecutive patients for whom laboratory tests were ordered.

A disadvantage of our method was the heterogeneity in terms of laboratory tests, abnormal results and diagnoses. This means that interpretation and management cannot be related to a specific test, abnormality or diagnosis. In the context of this study, however, it would have been unrealistic to reduce clinical variation to a minimum and thus force the physicians into a standardized study, since the primary goal was to examine whether pretest expectations influence interpretation, posttest probability estimates and management in day-to-day care. It could be interesting in future studies to examine in more detail how specific tests influence further diagnosis and management. Another limitation is that the abnormal results in the group of patients with a low pretest probability may have been less abnormal than those for patients with a high pretest probability. Such differences in the level of abnormality of test results may have influenced physicians' interpretation of the test results and their further management. This influence was difficult to correct for in our analyses, as many different laboratory tests were ordered. Future studies may address more specifically the influence of the level of abnormality of test results on interpretation and further management.

Finally, as each physician included several patients, there may have been a certain clustering of specific interpretations and behaviour at the level of the physician. We have not analyzed this, as the focus of the study was to explore how pretest expectations influence the interpretation of results at the level of individual patients, and we did not intend to explore the differences in interpretation between physicians. We recruited a large group of practitioners (87) to ensure external generalizability of our findings.

Our study found that the pretest disease probability strongly influenced the physicians' interpretation of the laboratory results and their posttest probability estimates. This seems in line with Bayesian theory, which shows that the significance of a particular test result depends on pretest probability [10]. But it has also been pointed out that physicians are often not very proficient at the calculations that this theory requires, and they do not routinely use these calculations [6,11]. There may be a gap between physicians' performance in terms of these calculations and the way they interpret laboratory results in routine care. This discrepancy might be addressed in future research.

Furthermore, the magnitude of abnormality of a test may be an important factor in the interpretation of results. When test results are dichotomized into normal and abnormal, important information may be lost. It may therefore be useful not to dichotomize test results, but to use, for example, likelihood ratios instead. These likelihood ratios may help physicians come to a more appropriate interpretation of test results [12], although it remains unclear if they are really helpful in routine practice [13,14].

Since many of the abnormal laboratory results hardly affected posttest probability estimates and management in our study, physicians should carefully consider if it was useful to order the tests in the first place. Also, the physicians ordered further investigations for nearly 10% of the patients for whom the original tests had been ordered for reassurance. It may be doubted if this was necessary, since it has been shown that investigations may also have negative consequences, such as an unjustified cascade of further investigations to explain unexpected abnormalities [15,16]. In view of the number of patients in this study who were offered further investigations, future research should examine how often negative consequences of laboratory testing, such as cascade processes, occur.

## Conclusions

Physicians' interpretation of laboratory results and further management after receiving the results of laboratory tests is clearly influenced by their pretest expectations. Physicians may use these expectations to seek a balance between over- and under-reacting to laboratory results. Our findings help to understand interpretation and use of laboratory results in day-to-day care. However, further research into the interpretation and use of laboratory results, including different levels of abnormality, is necessary and might in the future help to improve test interpretation and management.

## Competing interests

The authors declare that they have no competing interests.

## Authors' contributions

PHH, TvdW, RAGW and RPTMG designed the study. PHH, TvdW, RAGW participated in the data collection. PHH and BW analysed the data. PHH drafted the manuscript. RPTMG, TvdW, RAGW and BW participated in writing the manuscript. RPTMG supervised all parts of this study. All authors read and approved the final manuscript.

## Pre-publication history

The pre-publication history for this paper can be accessed here:

http://www.biomedcentral.com/1471-2296/11/13/prepub

## Supplementary Material

Additional file 1 **Questionnaire 1**. Questionnaire used for data recording when the physicians ordered the laboratory tests. Click here for file

Additional file 2 **Questionnaire 2**. Questionnaire used for data recording when the physicians received the test results. Click here for file

## References

[B1] Leurquin P Van Casteren V De Maeseneer J Use of blood tests in general practice: a collaborative study in eight European countries. Eurosentinel Study Group Br J Gen Pract 1995 45 390 21 25 7779470 PMC1239108

[B2] Weijden T van der van Bokhoven MA Dinant GJ van Hasselt CM Grol RP Understanding laboratory testing in diagnostic uncertainty: a qualitative study in general practice Br J Gen Pract 2002 52 485 974 980 12528582 PMC1314466

[B3] Phillips WR Thompson DJ Multi-channel laboratory testing and the unexpected abnormal result: a statistical myth corrected N Z Med J 1981 94 698 462 464 6950296

[B4] Mold JW Aspy CB Lawler FH Outcomes of an insurance company-sponsored multichannel chemistry screening initiative J Fam Pract 1998 47 2 110 117 9722798

[B5] Brigden ML Heathcote JC Problems in interpreting laboratory tests. What do unexpected results mean? Postgrad Med 2000 107 7 145 146 10.3810/pgm.2000.06.1127 10887452

[B6] Reid MC Lane DA Feinstein AR Academic calculations versus clinical judgments: practicing physicians' use of quantitative measures of test accuracy Am J Med 1998 104 4 374 380 10.1016/S0002-9343(98)00054-0 9576412

[B7] Verstappen WH ter Riet G Dubois WI Winkens R Grol RP Weijden T van der Variation in test ordering behaviour of GPs: professional or context-related factors? Fam Pract 2004 21 4 387 395 10.1093/fampra/cmh408 15249527

[B8] Holtgrave DR Lawler F Spann SJ Physicians' risk attitudes, laboratory usage, and referral decisions: the case of an academic family practice center Med Decis Making 1991 11 2 125 130 10.1177/0272989X9101100210 1865781

[B9] Houben PHH Weijden T van der van Bokhoven MA Droog A Winkens RA Grol R Overwegingen van huisartsen bij het interpreteren van laboratoriumonderzoek; een kwalitatief onderzoek Huisarts Wet 2005 48 7 736 732

[B10] Gigerenzer G Hoffrage U How to improve bayesian reasoning without instruction: frequency formats Psychol Rev 1995 102 4 684 704 10.1037/0033-295X.102.4.684

[B11] Steurer J Fischer JE Bachmann LM Koller M ter Riet G Communicating accuracy of tests to general practitioners: a controlled study BMJ 2002 324 7341 824 826 10.1136/bmj.324.7341.824 11934776 PMC100792

[B12] Brown MD Reeves MJ Evidence-based emergency medicine/skills for evidence-based emergency Ann Emerg Med 2003 42 2 292 7 10.1067/mem.2003.274 12883521

[B13] Puhan MA Steurer J Bachmann LM ter Riet G A randomized trial of ways to describe test accuracy: the effect on physicians' post-test probability estimates Ann Intern Med 2005 143 3 184 189 16061916 10.7326/0003-4819-143-3-200508020-00004

[B14] Sonis J How to use and interpret interval likelihood ratios Fam Med 1999 31 6 432 7 10367208

[B15] Deyo RA Cascade effects of medical technology Annu Rev Public Health 2002 23 23 44 10.1146/annurev.publhealth.23.092101.134534 11910053

[B16] Vafiadis P The dilemma of the unexpected result Aust Fam Physician 1996 25 6 971 973 8687319

