# Supplementary material for: Pretest expectations strongly influence interpretation of abnormal laboratory results and further management
Source: BMC Fam Pract. 2010 Feb 16;11:13. doi: 10.1186/1471-2296-11-13 (PMC2829524; doi:10.1186/1471-2296-11-13)
Supplement: Additional file 1 — Questionnaire 1. Questionnaire used for data recording when the physicians ordered the laboratory tests. [file 1471-2296-11-13-S1.DOC]

| **General Data** | | | | | | | | | | |
| --- | --- | --- | --- | --- | --- | --- | --- | --- | --- | --- |
|  | | | | | | | | | | |
| *1.* | *Date of consultation?* | |  | | | *.. .. - .. .. - 20 .. ..* | | | | |
| *2.* | *Patient’s date of birth?* | |  | | | .. .. - .. .. - .. .. .. .. | | | | |
| *3.* | *Patient’s sex* | |  | | | male O female O | | | | |
|  | | | | | | | | | | |
| **Reason for ordering lab test(s)** | | | | | | | | | | |
|  | | | | | | | | | | |
| 4. | *Why are you ordering this test / these tests?* Please check one box in the first column and one in the second column. There are no correct or incorrect answers. | | | | | | | | | |
|  |  |  | |  | | | | |  | |
|  |  |  | | most important reason | | | | | second reason | |
|  | 1. | Excluding a specific disease | | | | O^1^ | | | | O^1^ |
|  | 2. | Establishing/confirming a diagnosis | | | | O^2^ | | | | O^2^ |
|  | 3. | At patient’s request | | | | O^3^ | | | | O^3^ |
|  | 4. | To reassure the patient | | | | O^4^ | | | | O^4^ |
|  | 5. | Because I’m not sure what is the matter | | | | O^5^ | | | | O^5^ |
|  | 6. | To help me decide on treatment | | | | O^6^ | | | | O^6^ |
|  | 7. | As a check-up for the patient’s known condition | | | | O^7^ | | | | O^7^ |
|  | 8. | To screen for diabetes, hypertension or cholesterol | | | | O^8^ | | | | O^8^ |
|  | 9. | Other, namely  ........................................................... | | | | O^9^ | | | | O^9^ |
|  |  |  | | | |  | | | | |
| **Please read this!**  If you have only indicated reasons 7 and/or 8, you can now **stop** completing this questionnaire. | | | | | | | | | | |
|  |  | | | | | | | | | |
| **The diagnosis** | | | | | | | | | | |
|  |  |  | | | | | |  | | |
|  | The following questions relate to **all diagnoses** you are currently considering for this patient. | | | | | | | | | |
|  |  |  | | | | | |  | | |
| *5.a.* | *Do you suspect that the patient has a somatic disorder?* | | | | | | | | | |
|  |  |  | | | |  | | | | |
|  |  | Definitely not | | | | O | | | | |
|  |  | Probably not | | | | O | | | | |
|  |  | Maybe | | | | O | | | | |
|  |  | Probably yes | | | | O | | | | |
|  |  | Definitely yes | | | | O | | | | |
|  |  |  | | | |  | | | | |
| *5.b.* | Do you suspect an **innocuous** somatic disorder? | | | | | | | | | |
|  |  |  | | | |  | | | | |
|  |  | Definitely not | | | | O | | | | |
|  |  | Probably not | | | | O | | | | |
|  |  | Maybe | | | | O | | | | |
|  |  | Probably yes | | | | O | | | | |
|  |  | Definitely yes | | | | O | | | | |
|  |  | | | | | | | | | |
| *5.c.* | *Do you suspect a* ***serious*** *somatic disorder?* | | | | | | | | | |
|  |  |  | | | |  | | | | |
|  |  | Definitely not | | | | O | | | | |
|  |  | Probably not | | | | O | | | | |
|  |  | Maybe | | | | O | | | | |
|  |  | Probably yes | | | | O | | | | |
|  |  | Definitely yes | | | | O | | | | |
|  |  |  | | | |  | | | | |
| *6.* | *What is currently your most probable diagnosis or working hypothesis?*  …………………………………………………………………………………………………………. | | | | | | | | | |
|  |  |  | | | |  | | | | |
| *7.a.* | *Are you considering any other diagnoses?* | | | | | O  O | No  Yes | | | |
|  |  |  | | | |  |  | | | |
| *7.b.* | *If so, which one(s)?*  *1. ...............................................................................................................................................*  *2. ...............................................................................................................................................*  *3. ...............................................................................................................................................*  *4. ...............................................................................................................................................* | | | | | | | | | |
|  |  |  | | | | | |  | | |
|  |  |  | | | | | |  | | |
| **Management** | | | | | | | | | | |
|  |  |  | | | | | |  | | |
| *8.* | *What will be your management until the test results become available?* Please indicate the main components of your management, by ticking one box in the first column and one in the second. | | | | | | | | | |
|  |  |  | | |  | | | |  | |
|  |  |  | | | policy 1 | | | | policy 2 | |
|  | 1. | Reassuring, explaining | | | | O^1^ | | | | O^1^ |
|  | 2. | Expectative, wait-and-see | | | | O^2^ | | | | O^2^ |
|  | 3. | Advice (on lifestyle, diet, complaints, etc.) | | | | O^3^ | | | | O^3^ |
|  | 4. | Instructions (when to contact me, etc.) | | | | O^4^ | | | | O^4^ |
|  | 5. | New/follow-up appointment (telephone, consultation, home visit, etc.) | | | | O^5^ | | | | O^5^ |
|  | 6. | Medication (start, stop, adjust) | | | | O^6^ | | | | O^6^ |
|  | 7. | Referral or consultation (specialist, therapist, etc.) | | | | O^7^ | | | | O^7^ |
|  | 8. | Other, namely  ................................................... | | | | O^8^ | | | | O^8^ |
|  |  |  | | | |  | | | | |
|  |  |  | | | |  | | | | |
|  |  |  | | | |  | | | | |
|  |  |  | | | |  | | | | |
| **Thank you very much for cooperating with this study!** | | | | | | | | | | |
