# Supplementary material for: Pretest expectations strongly influence interpretation of abnormal laboratory results and further management
Source: BMC Fam Pract. 2010 Feb 16;11:13. doi: 10.1186/1471-2296-11-13 (PMC2829524; doi:10.1186/1471-2296-11-13)
Supplement: Additional file 2 — Questionnaire 2. Questionnaire used for data recording when the physicians received the test results. [file 1471-2296-11-13-S2.DOC]

| **General data** | | | | | | | | | |
| --- | --- | --- | --- | --- | --- | --- | --- | --- | --- |
|  | | | | | | | | | |
| *1.* | *Current date?* | |  | | *.. .. - .. .. - 20 .. ..* | | | | |
| *2.* | *Patient’s date of birth?* | |  | | .. .. - .. .. - .. .. .. .. | | | | |
| *3.* | *Patient’s sex?* | |  | | male O female O | | | | |
|  | | | | | | | | | |
| **Please read this!**  When answering the questions below, **please disregard** any test results relating to check-ups for the patient’s known conditions or to screening for hypertension, diabetes or cholesterol levels. If the test results only relate to these items, please **do not continue** completing this questionnaire. | | | | | | | | | |
|  | | | | | | | | | |
| **Test result** | | | | | | | | | |
|  | | | | | | | | | |
| *4.* | *How would you* ***interpret*** *this lab test results for this patient?* Please tick one box. If you prefer to tick multiple boxes please select the most relevant one. | | | | | | | | |
|  |  |  | | |  | | | |  |
|  |  |  | | |  | | | |  |
|  | *I interpret this test results for this patient as:* | | | |  | | | |  |
|  |  |  | | |  | | | |  |
|  |  | Clearly abnormal | | | O | | | |  |
|  |  | Possibly abnormal | | | O | | | |  |
|  |  | Normal | | | O | | | |  |
|  |  |  | | |  | | | | |
| **The diagnosis** | | | | | | | | | |
|  |  |  | | | | |  | | |
|  | The following questions relate to **all diagnoses** you are now considering for this patient. | | | | | | | | |
|  |  |  | | | | |  | | |
| *5.a.* | *Do you now suspect a somatic disorder?* | | | | | | | | |
|  |  |  | | |  | | | | |
|  |  | Definitely not | | | O | | | | |
|  |  | Probably not | | | O | | | | |
|  |  | Maybe | | | O | | | | |
|  |  | Probably yes | | | O | | | | |
|  |  | Definitely yes | | | O | | | | |
|  |  |  | | |  | | | | |
| *5.b.* | Do you now suspect an **innocuous** somatic disorder? | | | | | | | | |
|  |  |  | | |  | | | | |
|  |  | Definitely not | | | O | | | | |
|  |  | Probably not | | | O | | | | |
|  |  | Maybe | | | O | | | | |
|  |  | Probably yes | | | O | | | | |
|  |  | Definitely yes | | | O | | | | |
|  |  | | | | | | | | |
|  |  | | | | | | | | |
|  |  | | | | | | | | |
|  |  | | | | | | | | |
|  |  | | | | | | | | |
|  |  | | | | | | | | |
|  |  | | | | | | | | |
|  |  | | | | | | | | |
|  |  | | | | | | | | |
| *5.c.* | *Do you now suspect a* ***serious*** *somatic disorder?* | | | | | | | | |
|  |  |  | | |  | | | | |
|  |  | Definitely not | | | O | | | | |
|  |  | Probably not | | | O | | | | |
|  |  | Maybe | | | O | | | | |
|  |  | Probably yes | | | O | | | | |
|  |  | Definitely yes | | | O | | | | |
|  |  |  | | |  | | | | |
| *6.* | *What is now your most probable diagnosis or working hypothesis?*  …………………………………………………………………………………………………………. | | | | | | | | |
|  |  |  | | |  | | | | |
| *7.a.* | *Are you now considering any other diagnoses?* | | | | O  O | No  Yes | | | |
|  |  |  | | |  |  | | | |
| *7.b.* | *If so, which one(s)?*  *1. ...............................................................................................................................................*  *2. ...............................................................................................................................................*  *3. ...............................................................................................................................................*  *4. ...............................................................................................................................................* | | | | | | | | |
|  |  |  | | | | |  | | |
|  |  |  | | | | |  | | |
| **Management** | | | | | | | | | |
|  |  |  | | | | |  | | |
| *8.* | *What is your management now?* Please indicate the main components of your management, by ticking one box in the first column and one in the second. | | | | | | | | |
|  |  |  | |  | | | |  | |
|  |  |  | | policy 1 | | | | policy 2 | |
|  | 1. | Reassuring, explaining | | | O^1^ | | | | O^1^ |
|  | 2. | Expectative, wait-and-see | | | O^2^ | | | | O^2^ |
|  | 3. | Advice (on lifestyle, diet, complaints, etc.) | | | O^3^ | | | | O^3^ |
|  | 4. | Further investigations (laboratory-, imaging-, etc.) | | | O^4^ | | | | O^4^ |
|  | 5. | Instructions (when to contact me, etc.) | | | O^5^ | | | | O^5^ |
|  | 6. | New/follow-up appointment (telephone, consultation, home visit, etc.) | | | O^6^ | | | | O^6^ |
|  | 7. | Medication (start, stop, adjust) | | | O^7^ | | | | O^7^ |
|  | 8. | Referral or consultation (specialist, therapist, etc.) | | | O^8^ | | | | O^8^ |
|  | 9. | Other, namely  ................................................... | | | O^9^ | | | | O^9^ |
|  |  |  | | |  | | | | |
|  |  |  | | |  | | | | |
| **Thank you very much for cooperating with this study!** | | | | | | | | | |
